# Furmonertinib, a Third-Generation EGFR Tyrosine Kinase Inhibitor, Overcomes Multidrug Resistance through Inhibiting ABCB1 and ABCG2 in Cancer Cells

**DOI:** 10.3390/ijms241813972

**Published:** 2023-09-12

**Authors:** Chung-Pu Wu, Yen-Ching Li, Megumi Murakami, Sung-Han Hsiao, Yun-Chieh Lee, Yang-Hui Huang, Yu-Tzu Chang, Tai-Ho Hung, Yu-Shan Wu, Suresh V. Ambudkar

**Affiliations:** 1 Graduate Institute of Biomedical Sciences, College of Medicine, Chang Gung University, Taoyuan 33302, Taiwan; 2 Department of Physiology and Pharmacology, College of Medicine, Chang Gung University, Taoyuan 33302, Taiwan; 3 Molecular Medicine Research Center, College of Medicine, Chang Gung University, Taoyuan 33302, Taiwan; 4 Department of Obstetrics and Gynecology, Taipei Chang Gung Memorial Hospital, Taipei 10507, Taiwan; 5 Laboratory of Cell Biology, Center for Cancer Research, National Cancer Institute, NIH, Bethesda, MD 20892, USA; 6 Department of Medicine, College of Medicine, Chang Gung University, Taoyuan 33302, Taiwan; 7 Department of Obstetrics and Gynecology, Keelung Chang Gung Memorial Hospital, Keelung 20401, Taiwan; 8 Department of Chemistry, Tunghai University, Taichung 40704, Taiwan

**Keywords:** ABC transporter, ABCB1, ABCG2, multidrug resistance, drug repurposing, furmonertinib

## Abstract

ATP-binding cassette transporters, including ABCB1 (P-glycoprotein) and ABCG2 (BCRP/MXR/ABCP), are pivotal in multidrug resistance (MDR) development in cancer patients undergoing conventional chemotherapy. The absence of approved therapeutic agents for multidrug-resistant cancers presents a significant challenge in effectively treating cancer. Researchers propose repurposing existing drugs to sensitize multidrug-resistant cancer cells, which overexpress ABCB1 or ABCG2, to conventional anticancer drugs. The goal of this study is to assess whether furmonertinib, a third-generation epidermal growth factor receptor tyrosine kinase inhibitor overcomes drug resistance mediated by ABCB1 and ABCG2 transporters. Furmonertinib stands out due to its ability to inhibit drug transport without affecting protein expression. The discovery of this characteristic was validated through ATPase assays, which revealed interactions between furmonertinib and ABCB1/ABCG2. Additionally, in silico docking of furmonertinib offered insights into potential interaction sites within the drug-binding pockets of ABCB1 and ABCG2, providing a better understanding of the underlying mechanisms responsible for the reversal of MDR by this repurposed drug. Given the encouraging results, we propose that furmonertinib should be explored as a potential candidate for combination therapy in patients with tumors that have high levels of ABCB1 and/or ABCG2. This combination therapy holds the potential to enhance the effectiveness of conventional anticancer drugs and presents a promising strategy for overcoming MDR in cancer treatment.

## 1. Introduction

The occurrence of multidrug resistance (MDR) presents a significant challenge in cancer treatment. MDR refers to cancer cells acquiring simultaneous resistance to multiple chemotherapeutic drugs that are structurally unrelated [1,2]. While the causes of MDR in cancer are complex and involve multiple factors, it is commonly linked to the overexpression of two well-studied and characterized ATP-binding cassette (ABC) transporters ABCB1 (P-glycoprotein; MDR1) and ABCG2 (BCRP; MXR; ABCP) [1,2]. These transporters actively remove a wide range of chemotherapeutic drugs from cancer cells, including commonly prescribed cytotoxic anticancer drugs, through ATP hydrolysis [2,3]. Vinca alkaloids, anthracyclines, taxanes, and mitoxantrone are among the well-known substrate drugs of ABCB1 and/or ABCG2 [1,2]. As a result, the overexpression of ABCB1 and ABCG2 has been linked to unfavorable prognosis and treatment failure [1,2] in individuals diagnosed with hematologic malignancies [4,5,6,7] and solid tumors [8,9,10]. Additionally, due to their prominent expression at critical blood-tissue barriers like the intestinal epithelium and the blood-brain barrier (BBB), ABCB1 and ABCG2 exert a considerable influence on the bioavailability and tissue distribution of these substrate drugs [1,2]. Consequently, the identification of potent inhibitors for ABCB1 and ABCG2 holds significant clinical importance.

At present, no clinically approved inhibitors of ABCB1 or ABCG2 exist for combating multidrug-resistant cancers, primarily due to unexpected adverse drug reactions [2,11,12]. The slow progress in developing synthetic agents to reverse MDR for clinical use has led researchers, including us, to shift their focus towards identifying already approved therapeutic drugs that could be repurposed (also referred to as drug repositioning or therapeutic switching) to sensitize multidrug-resistant cancer cells to conventional cytotoxic anticancer drugs [13,14,15]. In this study, we investigate the potential of repurposing furmonertinib, an epidermal growth factor receptor (EGFR) tyrosine kinase inhibitor (TKI), as a candidate for reversing MDR mediated by ABCB1 and ABCG2 in multidrug-resistant cancer cells. Furmonertinib (alflutinib, AST2818) is an irreversible EGFR TKI approved in China in 2021 to treat patients with locally advanced or metastatic non-small cell lung cancer (NSCLC) with confirmed EGFR T790M mutations, whose disease has progressed during or after EGFR TKI therapy [16]. In this study, we found that neither ABCB1 nor ABCG2 confers significant resistance to furmonertinib in cancer cells. Moreover, we discovered that furmonertinib effectively inhibits the drug efflux function of ABCB1 and ABCG2, leading to a substantial restoration of drug-induced apoptosis and reversal of MDR in cancer cells overexpressing these transporters. Our data show that furmonertinib exhibits comparable inhibitory activity against both ABCB1 and ABCG2. The considerable pharmacological effectiveness of furmonertinib in combating transporter-mediated MDR should encourage further exploration of combination therapy trials for patients with multidrug-resistant cancers. These findings hold promising implications for overcoming resistance and enhancing the effectiveness of cancer treatment.

## 2. Results

### 2.1. Furmonertinib Exhibits Equal Sensitivity in Both Drug-Sensitive and Multidrug-Resistant Cells Overexpressing ABCB1 or ABCG2

Given that some EGFR inhibitors are known to be transport substrates of ABCB1 and/or ABCG2 [17,18], we conducted a comparison of the sensitivity of several paired parental cell lines and multidrug-resistant sublines to furmonertinib. The results, presented in Table 1, revealed that the ABCB1-overexpressing human ovarian cancer cell line NCI-ADR-RES, the ABCB1-overexpressing human epidermal cancer cell line KB-V1, the ABCG2-overexpressing human colon cancer cell line S1-MI-80, and the ABCG2-overexpressing human NSCLC cell line H460-MX20, as well as their corresponding drug-sensitive parental cell lines showed equal sensitivity to furmonertinib. To validate our findings, we further examined the cytotoxicity of furmonertinib in pcDNA3.1-HEK293, ABCB1-transfected MDR19-HEK293, and ABCG2-transfected R482-HEK293 cells. The resistance factor (RF) value, indicating the extent of cellular resistance to furmonertinib mediated by ABCB1 or ABCG2, was determined by dividing the IC_50_ values of furmonertinib in the multidrug-resistant subline by the IC_50_ values of furmonertinib in the respective parental lines (Table 1). In conclusion, our study demonstrates that furmonertinib exhibits equal cytotoxicity in all tested HEK293 transfectants, regardless of ABCB1 or ABCG2 overexpression.

### 2.2. Furmonertinib Reverses Multidrug Resistance Mediated by ABCB1 and ABCG2

**Figure 1:**
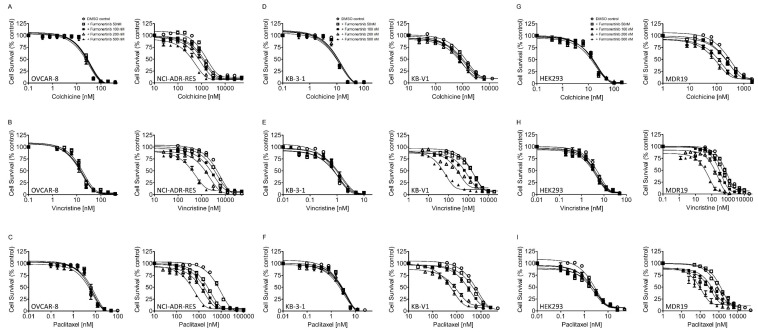
Furmonertinib enhances the sensitivity of ABCB1-overexpressing multidrug-resistant cells to cytotoxic drug substrates. The experiments were conducted using (**A**–**C**) OVCAR-8 (left) and NCI-ADR-RES (right) cancer cells, (**D**–**F**) KB-3-1 (left) and KB-V1 (right) cancer cells, as well as (**G**–**I**) pcDNA3.1-HEK293 (left) and MDR19-HEK293 (right) cells. The cells were treated with increasing concentrations of colchicine, vincristine, or paclitaxel in the presence of DMSO (open circles) or different concentrations of furmonertinib (50 nM, 100 nM, 200 nM, or 500 nM) for 72 h. Analysis was performed as described in Materials and Methods. The points represent the mean values obtained from at least three independent experiments, and the bars represent the SEM.

**Figure 2:**
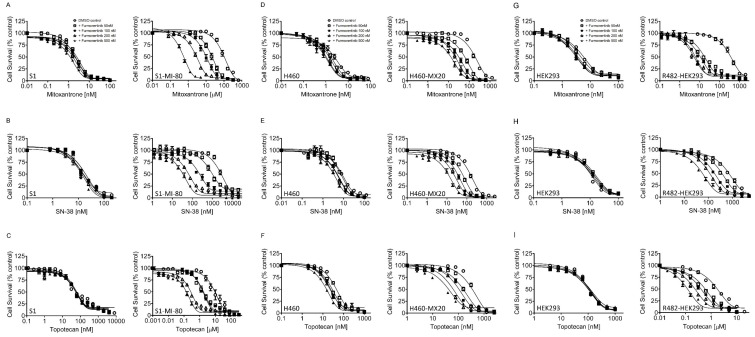
Furmonertinib enhances the sensitivity of multidrug-resistant cells overexpressing ABCG2 to cytotoxic drug substrates. (**A**–**C**) S1 (left) and S1-MI-80 (right) cancer cells, (**D**–**F**) H460 (left) and H460-MX20 (right) cancer cells, as well as (**G**–**I**) pcDNA3.1-HEK293 (left) and R482-HEK293 (right) cells were exposed to increasing concentrations of mitoxantrone, SN-38, or topotecan in the presence of either DMSO (open circles) or different concentrations of furmonertinib (50 nM, 100 nM, 200 nM, or 500 nM) for 72 h, followed by analysis as described in Materials and Methods. The data points represent the mean values obtained from at least three independent experiments, with the error bars indicating the SEM.

Being aware that numerous EGFR inhibitors had been previously discovered as modifiers of ABCB1 and/or ABCG2 [15,19], we explored the potential of furmonertinib to enhance chemosensitivity in multidrug-resistant cancer cells that overexpress ABCB1 or ABCG2. Our data revealed that furmonertinib effectively resensitized NCI-ADR-RES (Figure 1A–C) and KB-V1 (Figure 1D–F) cancer cells, as well as MDR19-HEK293 cells (Figure 1G–I), to ABCB1 substrates colchicine, vincristine, and paclitaxel in a concentration-dependent manner. Furthermore, we noted that furmonertinib exhibited a significant resensitization effect on S1-MI-80 (Figure 2A–C), H460-MX20 (Figure 2D–F), and R482-HEK293 cells (Figure 2G–I), making them more responsive to ABCG2 substrates, mitoxantrone, SN-38, and topotecan, in a concentration-dependent manner. Table 2 and Table 3 summarize the IC_50_ values and the extent of chemosensitization by furmonertinib, presented as the fold-reversal (FR) value. The FR value was determined as the ratio of the IC_50_ value for a cytotoxic drug when administered without an inhibitor to the IC_50_ value of the same cytotoxic drug when administered with the identical inhibitor in the corresponding cell line. In this study, 1 μM tariquidar and 1 μM Ko143 were employed as reference inhibitors for ABCB1 and ABCG2, respectively.

### 2.3. Furmonertinib Enhances Drug-Induced Apoptosis in Multidrug-Resistant Cancer Cells That Overexpress ABCB1 or ABCG2

**Figure 3:**
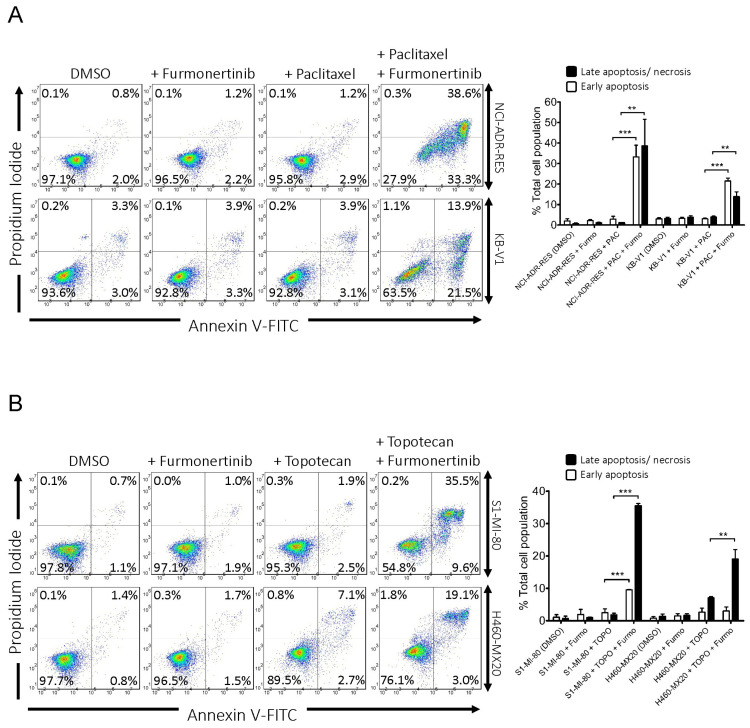
Furmonertinib augments apoptosis induced by cytotoxic drugs in ABCB1- and ABCG2-overexpressing multidrug-resistant cancer cells. In the case of (**A**) NCI-ADR-RES and KB-V1 cancer cells, treatment options included DMSO (control), 2 μM furmonertinib (+ Furmo), 1 μM paclitaxel (+ PAC) alone, or a combination of 1 μM paclitaxel and 2 μM furmonertinib (+ PAC + Furmo). For (**B**) S1-MI-80 and H460-MX20 cancer cells, the treatment options included DMSO (control), 2 μM furmonertinib (+ Furmo), 10 μM topotecan (+ TOPO) alone, or a combination of 10 μM topotecan and 2 μM furmonertinib (+ TOPO + Furmo). These treatments were applied for 48 h as described in Materials and Methods. Subsequently, Annexin V-FITC and PI staining were performed, and flow cytometry was used for analysis. The left side shows representative dot plots, while the right side presents quantified values as mean ± SD, calculated from at least three independent experiments. ** *p* < 0.01; *** *p* < 0.001, compared to the same treatment without furmonertinib.

The impact of furmonertinib on the paclitaxel-induced apoptosis was examined in NCI-ADR-RES and KB-V1 cancer cells, while the topotecan-induced apoptosis was investigated in S1-MI-80 and H460-MX20 cancer cells. The cells were subjected to Annexin V-FITC and propidium iodide (PI) staining, following the procedure described in Materials and Methods. NCI-ADR-RES and KB-V1 cells (Figure 3A) were treated with DMSO (control), 2 μM furmonertinib, 1 μM paclitaxel, or a combination of 1 μM paclitaxel and 2 μM furmonertinib for 48 h. The results revealed that furmonertinib significantly increased the paclitaxel-induced apoptotic cell population (from 4% to 72% in NCI-ADR-RES and from 7% to 35% in KB-V1). Similarly, in S1-MI-80 and H460-MX20 cells (Figure 3B), the treatment conditions were DMSO (control), 2 μM furmonertinib, 10 μM topotecan, or a combination of 10 μM topotecan and 2 μM furmonertinib for 48 h. Furmonertinib also significantly increased the topotecan-induced apoptotic cell population (from 4% to 45% in S1-MI-80 and from 10% to 22% in H460-MX20). These findings indicate that furmonertinib effectively enhances the apoptotic effect of substrate drugs of ABCB1 and ABCG2 in multidrug-resistant cancer cells by inhibiting the activity of these transporters.

### 2.4. Furmonertinib Attenuates the Drug Transport Function of ABCB1 and ABCG2

**Figure 4:**
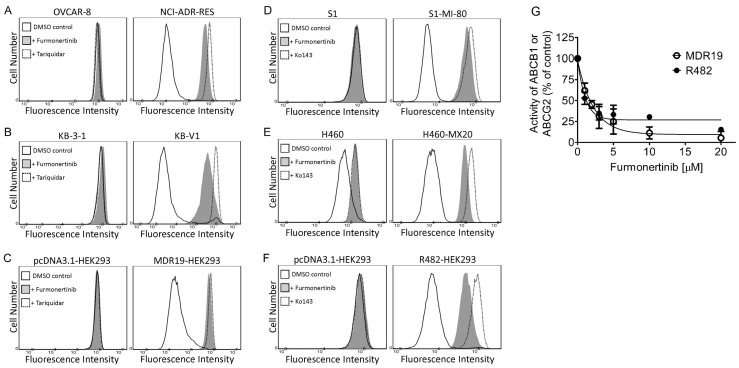
Furmonertinib diminishes the drug efflux activity of ABCB1 and ABCG2. The intracellular accumulation of calcein in (**A**) OVCAR-8 and NCI-ADR-RES, (**B**) KB-3-1 and KB-V1, (**C**) pcDNA3.1-HEK293 and MDR19-HEK293 cells, or PhA in (**D**) S1 and S1-MI-80, (**E**) H460 and H460-MX20, and (**F**) pcDNA3.1-HEK293 and R482-HEK293 cells was evaluated after treatment with DMSO (control, solid lines), 5 μM furmonertinib (dotted lines), 5 μM tariquidar or Ko143 (shaded, solid lines). (**G**) The concentration-dependent inhibition of ABCB1-mediated calcein-AM efflux in MDR19-HEK293 cells and ABCG2-mediated PhA in R482-HEK293 cells by furmonertinib was analyzed. The results include representative histograms, and the quantification values are presented as mean ± SD, calculated from at least three independent experiments. The fluorescence signal was analyzed following the protocol described in Materials and Methods.

A predominant approach for a modulator to overcome MDR mediated by ABCB1 and ABCG2 involves directly impeding the activity of ABCB1 and ABCG2 within multidrug-resistant cancer cells [11,14,15,19,20]. Therefore, we conducted a short-term fluorescent drug accumulation assay to determine the effect of furmonertinib on the function of ABCB1 and ABCG2. As anticipated, we observed a marked decrease in the intracellular accumulation of fluorescent calcein within the cell lines overexpressing ABCB1 in comparison to their respective drug-sensitive parental cell lines (Figure 4A–C). Treatment with 5 μM furmonertinib led to a significant increase in the intracellular accumulation of calcein in ABCB1-overexpressing NCI-ADR-RES (Figure 4A), KB-V1 (Figure 4B), and MDR19-HEK293 (Figure 4C) cells. Similarly, in the ABCG2-overexpressing cell lines, we observed a decreased intracellular accumulation of the known fluorescent ABCG2 substrate pheophorbide A (PhA) compared to the drug-sensitive parental cell lines (Figure 4D–F). Upon treatment with 5 μM furmonertinib, the intracellular accumulation of PhA was significantly increased in ABCG2-overexpressing S1-MI-80 (Figure 4D), H460-MX20 (Figure 4E), and R482-HEK293 (Figure 4F) cells. Next, to assess the relative efficacy of furmonertinib in relation to the functioning of ABCB1 and ABCG2, the concentration-dependent inhibition of furmonertinib on ABCB1-mediated calcein-AM efflux and ABCG2-mediated PhA efflux was determined in MDR19-HEK293 and R482-HEK293 cells (Figure 4G). The findings revealed that furmonertinib blocked ABCB1 activity in MDR19-HEK293 cells with a calculated IC_50_ value of 1.53 ± 0.08 μM. Furthermore, furmonertinib inhibited ABCG2 activity in R482-HEK293 cells with a calculated IC_50_ value of 2.12 ± 0.64 μM. In these experiments, 5 μM tariquidar and 5 μM Ko143 served as positive controls for complete inhibition of the activity of ABCB1 and ABCG2, respectively.

### 2.5. Furmonertinib Does Not Alter the protein Expression of ABCB1 and ABCG2 in Multidrug-Resistant Cancer Cells

**Figure 5:**
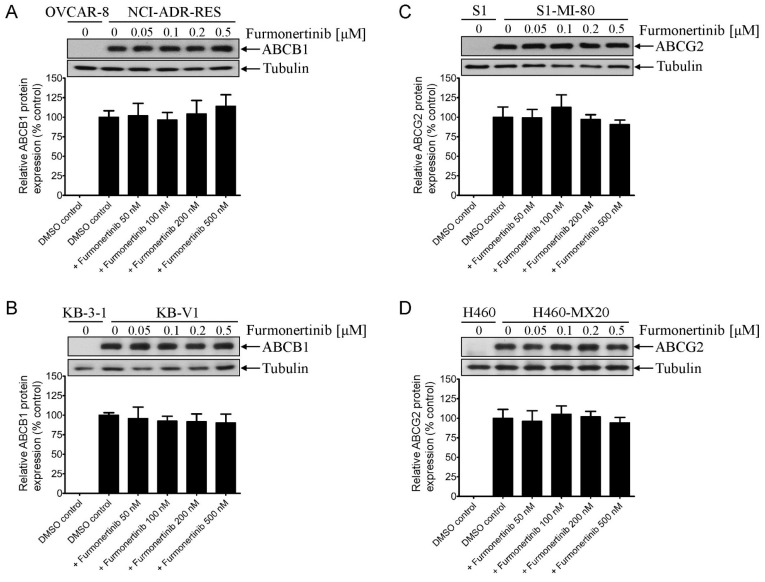
Effect of furmonertinib on the protein expression of ABCB1 or ABCG2 in multidrug-resistant cancer cells. The ABCB1-overexpressing (**A**) NCI-ADR-RES and (**B**) KB-V1, and ABCG2-overexpressing (**C**) S1-MI-80 and (**D**) H460-MX20 cancer cells were treated with DMSO (vehicle control) or furmonertinib at 50 nM, 100 nM, 200 nM, or 500 nM for 72 h before the cell lysates were processed for Western blotting, as described in Materials and Methods. The upper panels represent representative immunoblots showing human ABCB1 or ABCG2 protein, along with the internal loading control α-tubulin. The lower panels show the corresponding quantification values. All data are presented as mean ± SD, calculated from at least three independent experiments.

Recognizing that inducing a transient downregulation in the protein expression of ABCB1 and ABCG2 through drug treatment could also restore sensitivity to anticancer drugs in multidrug-resistant cancer cells [21,22], we investigated how furmonertinib influences the protein expression of ABCB1 and ABCG2 in multidrug-resistant cancer cells. Our findings indicate that treatment with furmonertinib at concentrations ranging from 50 nM to 500 nM did not produce a significant impact on the protein expression of ABCB1 in NCI-ADR-RES (Figure 5A) and KB-V1 (Figure 5B) cells, nor on ABCG2 in S1-MI-80 (Figure 5C) and H460-MX20 (Figure 5D) cancer cells over a period of 72 h. These outcomes strongly suggest that the reversal of ABCB1- and ABCG2-mediated MDR by furmonertinib is achieved through antagonizing drug efflux functionality rather than by modifying the protein expression of ABCB1 and ABCG2.

### 2.6. Furmonertinib Stimulates the ATPase Activity of ABCB1 and ABCG2

**Figure 6:**
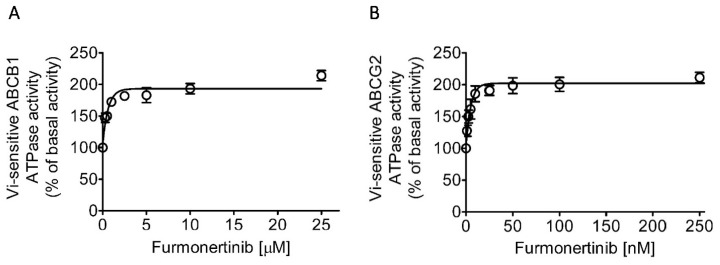
Furmonertinib exhibited a stimulatory effect on the ATPase activity of ABCB1 and ABCG2. The impact of furmonertinib on ATP hydrolysis, mediated by (**A**) ABCB1 and (**B**) ABCG2, was assessed using membrane vesicles derived from High-Five insect cells infected with recombinant baculovirus carrying the *MDR1* gene or *ABCG2* gene. The vanadate (Vi)-sensitive ATPase activity was measured following the procedure described in Materials and Methods. The basal ATPase activity in the membrane vesicles of non-transfected parental High-Five insect cells is very low, ranging from 3 to 5 nanomoles P_i_/min/mg protein. The data presented are the mean values obtained from at least three independent experiments, with error bars representing the standard deviation.

Subsequently, we assessed the ATPase activity of ABCB1 and ABCG2 in the presence of furmonertinib at various concentrations. The results depicted in Figure 6 demonstrate that furmonertinib stimulated the ATPase activity of both ABCB1 and ABCG2 in a concentration-dependent manner, with calculated EC_50_ values of approximately 0.6 μM (Figure 6A) and 3 nM (Figure 6B), respectively. These findings suggest that furmonertinib interacts with ABCB1 and ABCG2 at their respective substrate-binding sites.

### 2.7. Docking of Furmonertinib in the Drug-Binding Pockets of ABCB1 and ABCG2

**Figure 7:**
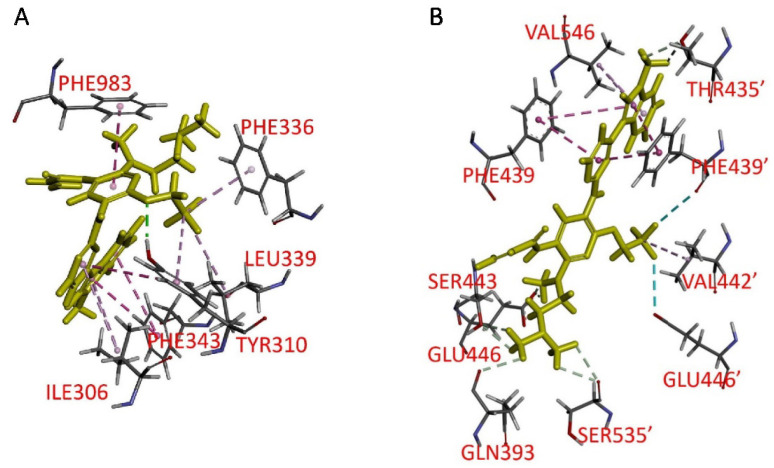
The docking of furmonertinib within the drug-binding regions of (**A**) ABCB1 (PDB: 6QEX) and (**B**) ABCG2 (PDB: 8BI0) was performed using BIOVIA Discovery Studio 4.0 software, following the method described in Materials and Methods. The lowest energy poses for the binding of furmonertinib are represented by molecular models shown in yellow, using a stick representation for the compound. The interacting amino acid residues are indicated by different colors, with carbon in gray, oxygen in red, nitrogen in blue, hydrogen in light gray, and sulfur in yellow. Proposed interactions between furmonertinib and the amino acid residues are depicted as dotted lines.

For a deeper understanding of how furmonertinib interacts with the drug-binding pockets of ABCB1 and ABCG2, we conducted molecular docking analysis in silico, utilizing the inward-open conformation of human ABCB1 (PDB:6QEX) [23] and ABCG2 (PDB: 8BI0) [24]. Our analysis revealed that the interactions between furmonertinib and ABCB1 were predominantly hydrophobic, with its aromatic rings forming interactions with Phe^343^ (indole), Ile^306^ (pyrimidine), and Phe^983^ (pyridine) residues. Additionally, the trifluoroethoxy moiety was predicted to form hydrophobic interactions with Phe^336^, Leu^339^, and Tyr^310^, with a hydrogen bond observed between Tyr^310^ and furmonertinib in the lowest energy docking pose (Figure 7A). Similarly, for ABCG2, the binding of furmonertinib was predicted to occur in the substrate/inhibitor binding pocket, with potential hydrophobic interactions between indole/pyrimidine and Phe^439^, Phe^439’^, and Val^546^, and between the CF_3_ moiety and Val^442’^ (Figure 7B).

## 3. Discussion

Osimertinib, the first FDA-approved third-generation EGFR TKI for EGFR T790M-positive NSCLC treatment [25], has been recognized for its ability to attenuate the drug efflux function of ABCB1 and ABCG2, thus reversing MDR mediated by both transporters [15,26]. Similar to osimertinib, furmonertinib is an EGFR TKI approved for EGFR T790M-positive NSCLC treatment [16]. While the preclinical data regarding furmonertinib inhibition of EGFR mutants (including T790M and ex20ins) have yet to be released by the manufacturer, recent studies have demonstrated that it is superior to gefitinib in terms of efficacy as a first-line treatment in patients with EGFR mutation-positive locally advanced or metastatic NSCLC [27], in lung adenocarcinoma patients harboring *EGFR*ex20ins with favorable response to furmonertinib as a single agent [28,29], and in combination with anlotinib [30]. Furmonertinib is currently being evaluated in combination trials for patients with EGFR mutation-positive NSCLC (ClinicalTrials.gov Identifier: NCT05430802, NCT05503667, NCT05334277, NCT04895930, and NCT03787992).

In terms of structure, furmonertinib shares a similar chemical backbone with osimertinib, but it features a pyridyl ring instead of a phenyl ring and replaces the methyl group with a trifluoromethyl group. Consequently, we investigated the interactions of furmonertinib with ABCB1 and ABCG2 in multidrug-resistant cancer cell lines. Since osimertinib had previously been identified as a substrate of both ABCB1 and ABCG2 [18], we assessed the susceptibility of various cancer cell lines to furmonertinib. As shown in Table 1, there was no notable difference in susceptibility to furmonertinib treatment between the drug-sensitive parental cell lines and the respective multidrug-resistant sublines overexpressing ABCB1 or ABCG2. Our data suggest that furmonertinib was not efficiently effluxed out of cancer cells by ABCB1 and ABCG2. While further investigation is required to understand the mechanisms of acquired resistance in patients treated with furmonertinib, our results indicate that the overexpression of ABCB1 or ABCG2 is unlikely to play a significant role in the development of resistance to furmonertinib in cancer cells. Subsequently, we investigated the ability of furmonertinib to reverse MDR mediated by ABCB1 and ABCG2. Our findings demonstrated that at sub-micromolar concentrations, furmonertinib sensitized multidrug-resistant cancer cells overexpressing ABCB1 or ABCG2, as well as cells with ectopic expression of human ABCB1 or ABCG2, to cytotoxic drugs in a concentration-dependent manner (Figure 1 and Figure 2). Notably, furmonertinib effectively restored the chemosensitivity of NCI-ADR-RES and KB-V1 cancer cells to vincristine and paclitaxel to a similar degree. However, its effect on ABCB1-mediated colchicine resistance in ABCB1-overexpressing cells was less pronounced. Furthermore, furmonertinib displayed comparable effects on enhancing paclitaxel-induced apoptosis in NCI-ADR-RES and KB-V1 cancer cells (Figure 3A), as well as enhancing topotecan-induced apoptosis in S1-MI-80 and H460-MX20 (Figure 3B) cancer cells. Additionally, when we evaluated the effect of furmonertinib on fluorescent drug transport mediated by ABCB1 and ABCG2, we observed a similar impact on ABCB1-mediated calcein-AM efflux and ABCG2-mediated PhA efflux in ABCB1- and ABCG2-overexpressing cells (Figure 4). These data align with the chemosensitizing results shown in Figure 1 and Figure 2. It is noteworthy that due to the presence of a trace amount of ABCG2 protein in the parental H460 cell line [31,32], we observed a noticeable chemosensitization effect by furmonertinib in H460 cancer cells (Table 3). Overall, our findings suggest that furmonertinib effectively countered MDR in cancer cells overexpressing ABCB1 or ABCG2, achieved through the inhibition of drug efflux activity for both transporters, rather than by inducing changes in the protein expression of ABCB1 and ABCG2.

**Figure 8:**
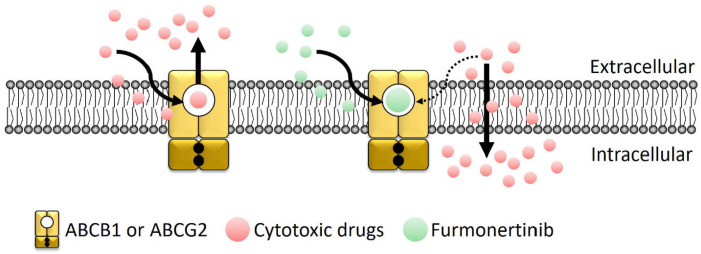
This simplified schematic illustrates how furmonertinib diminishes the drug efflux activity of ABCB1 and ABCG2, resulting in heightened sensitivity of multidrug-resistant cancer cells that overexpress ABCB1 and ABCG2 to cytotoxic anticancer drugs. In the absence of furmonertinib, substrate drugs (red circles) for ABCB1 and/or ABCG2 are actively removed by these transporters (yellow) within the cancer cells. However, in the presence of furmonertinib (green circles), it competes with the substrate drugs for the same drug-binding pocket on ABCB1 and ABCG2, thereby weakening their drug efflux function and facilitating the intracellular accumulation of cytotoxic drugs in the multidrug-resistant cancer cells.

The biochemical data provided further support for the interactions of furmonertinib with ABCB1 and ABCG2. Considering the established link between the ATPase activity of ABCB1 and ABCG2 and their overall function [33,34], the stimulatory effect of furmonertinib on ATP hydrolysis mediated by both transporters indicates that furmonertinib indeed engages with their substrate-binding sites (Figure 6). To explore potential binding sites within the drug-binding pockets of ABCB1 and ABCG2, in silico docking analysis of furmonertinib to the inward-open conformation of human ABCB1 and ABCG2 was conducted (Figure 7). The docking analysis revealed that furmonertinib was positioned in the central cavity between two transmembrane domains of ABCB1 and ABCG2. Furmonertinib was predicted to interact with several amino acid residues on helices 5, 6, and 12. Notably, strong pi–pi interactions were observed between the indole/pyrimidine rings of furmonertinib and the Phe^439^/Phe^439′^ residues in ABCG2. Additionally, furmonertinib exhibited an additional hydrophobic interaction between its aromatic rings and Val^546^ in ABCG2. Moreover, furmonertinib formed extra interactions with Val^442’^ through its trifluoroethoxy group, along with interactions between Val^546^ and its indole ring. These combined interactions led to a low-binding energy (−113 kcal/mol) for furmonertinib. Overall, despite the lack of an in vivo model to validate the findings, our results suggest that furmonertinib exerts its inhibitory effects on ABCB1 and ABCG2 by establishing interactions with multiple residues within their substrate-binding pockets, effectively displacing other substrate drugs from binding at the same site (Figure 8).

It is important to highlight that several clinical trials have provided evidence for the benefits of combination therapy involving a TKI and cytotoxic anticancer drugs as opposed to monotherapy. For instance, erlotinib has been shown to improve survival in patients with advanced pancreatic cancer treated with gemcitabine [35]. Similarly, lapatinib plus capecitabine have shown superiority over capecitabine alone in women with HER2-positive advanced breast cancer, who experienced disease progression after previous anthracycline, taxane, and trastuzumab therapy [36]. Recent findings from a phase I study revealed that the sequential administration of nilotinib prior to doxorubicin treatment led to a reduction in ABCB1 activity in sarcoma patients, without a corresponding increase in cardiotoxicity. This study concluded that co-administering nilotinib with doxorubicin is a viable approach [37]. Building upon these results, there is a rationale for exploring combination trials involving other TKIs and conventional cytotoxic anticancer agents, aiming to enhance cancer treatment.

In summary, our study revealed that furmonertinib functions as a modulator for both ABCB1 and ABCG2 transporters. It has the capability to sensitize multidrug-resistant cancer cells that overexpress ABCB1 and ABCG2 to cytotoxic anticancer drugs by attenuating their drug efflux function. Considering these outcomes, the potential for utilizing furmonertinib in combination therapies to overcome chemoresistance among patients whose tumors exhibit elevated expression of ABCB1 and/or ABCG2 merits further comprehensive exploration.

## 4. Materials and Methods

### 4.1. Chemical Reagents and Cell Culture

Furmonertinib was purchased from Selleckchem (Houston, TX, USA). The FITC Annexin V Apoptosis Detection Kit was obtained from BD Pharmingen (San Diego, CA, USA). The TOOLS Cell Counting (CCK-8) kit was obtained from Biotools Co., Ltd. (Taipei, Taiwan). Unless explicitly mentioned, all other chemicals were acquired from Sigma-Aldrich (St. Louis, MO, USA). KB-V1, an ABCB1-overexpressing human epidermal cancer cell line, and its drug-sensitive parental line KB-3-1 [38] were maintained in Dulbecco’s Modified Eagle Medium (DMEM) (Gibco, Invitrogen, Carlsbad, CA, USA). Human embryonic kidney 293 cell line transfected with either empty pcDNA3.1 vector (pcDNA3.1-HEK293), or human ABCB1 (MDR19-HEK293) [39], or human ABCG2 (R482-HEK293) [40] were sustained in DMEM, as well. The human NSCLC cell line H460 and its ABCG2-overexpressing subline H460-MX20 [41] were sustained in Rosewell Park Memorial Institute (RPMI-1640) medium (Gibco, Invitrogen, Carlsbad, CA, USA). The human colon cancer cell line S1 and its ABCG2-overexpressing subline S1-MI-80 [42] were also sustained in RPMI-1640 medium. The ABCB1-overexpressing human ovarian cancer cell line NCI-ADR-RES and its drug-sensitive parental line OVCAR-8 [43] were sustained in RPMI-1640 medium. Specific drug concentrations were added to the culture media for certain cell lines, KB-V1 cells received 1 μg/mL vinblastine [44], NCI-ADR-RES cells received 850 nM doxorubicin [43], H460-MX20 cells received 20 nM mitoxantrone [45], and S1-MI-80 cells received 80 μM mitoxantrone [42]. The HEK293 transfectants were sustained in a medium with the addition of G418 [39,40]. All cell lines were kept at 37 °C in a humidified chamber with 5% CO_2_ and cultured in media supplemented with 10% FBS (fetal bovine serum), L-glutamine, and a penicillin/streptomycin solution mixture (100 units/mL). The cell lines were generous gifts from Drs. Michael Gottesman and Susan Bates (NCI, NIH, Bethesda, MD, USA). Periodic screening for mycoplasma contamination was performed using a TOOLS Mycoplasma Detection Kit (Taipei, Taiwan), and the cells were maintained in a drug-free medium for 7 days before conducting the assays.

### 4.2. Cytotoxicity Assays

The cytotoxicity assays were conducted following a previously described method [19]. In brief, cells were seeded in 96-well flat-bottom plates and allowed to adhere overnight at 37 °C in a 5% CO_2_-humidified environment. Subsequently, the cells were exposed to various concentrations of furmonertinib or combinations of conventional anticancer agents and furmonertinib for an additional 72 h. Afterwards, the cells were processed using CCK-8 or MTT reagent, as described in previous work [20]. The IC50 values were determined by fitting a concentration-response curve obtained from at least three independent experiments. Furmonertinib was added to the cytotoxicity assays to assess the extent of reversal by furmonertinib, presented as the fold-reversal (FR) value following the methodology described earlier [20].

### 4.3. Apoptosis Assays

To evaluate the apoptotic effect of furmonertinib in multidrug-resistant cancer cell lines, we employed the Annexin V-FITC and PI staining method as previously described [46], following the manufacturer’s instructions from BD Pharmingen. In brief, the cells were subjected to various treatments, including DMSO (control), furmonertinib alone, paclitaxel or topotecan alone, or combinations of paclitaxel or topotecan with furmonertinib, as indicated. After 48 h of treatment, the cells were stained with 1.25 µg/mL Annexin V-FITC and 100 µg/mL PI at room temperature for 15 min. The labeled cells were then analyzed using a FACScan flow cytometer equipped with CellQuest software version 5.1, following the procedure described previously [15].

### 4.4. Fluorescent Drug Transport Assays

Cells were detached using trypsin and suspended in IMDM (Iscove’s Modified Dulbecco’s Medium) containing 5% FBS, along with the known ABCB1 substrate calcein-AM or the known ABCG2 substrate PhA. These suspensions were treated with DMSO, furmonertinib, tariquidar, or Ko143, following the procedure previously described [47]. To assess the intracellular accumulation of calcein and PhA, measured as fluorescence intensity, a BD Biosciences FACScan flow cytometer was utilized. The fluorescence intensity was detected at 485 nm excitation and 535 nm emission for calcein, and at 395 nm excitation and 670 nm emission for PhA. The data analysis was performed using BD Biosciences CellQuest software version 5.1 (Becton-Dickinson Biosciences, San Jose, CA, USA) and FlowJo software version 7.6.1 (Tree Star, Inc., Ashland, OR, USA), as described in previous work [47].

### 4.5. Immunoblot Analysis

Immunoblotting was carried out following the standard procedure as previously described [20]. In brief, cancer cells were treated with DMSO (control) or furmonertinib at concentrations of 20 nM, 100 nM, 200 nM, or 1000 nM for 72 h. After the treatment, the cells were harvested and subjected to SDS-polyacrylamide electrophoresis (SDS-PAGE). For Western blotting, the primary antibody C219 (diluted 1:3000, Merck Millipore, Burlington, MA, USA) was used to detect human ABCB1, the primary antibody BXP-21 (diluted 1:15,000, Abcam, Cambridge, MA, USA) was used to detect human ABCG2, and the primary antibody anti-alpha tubulin (diluted 1:100,000, Sigma-Aldrich, St. Louis, MO, USA) was used as a positive loading control for tubulin. A horseradish peroxidase-conjugated goat anti-mouse immunoglobulin G (IgG) (diluted 1:100,000, Abcam, Cambridge, MA, USA) was used as the secondary antibody. Signal detection was performed using the enhanced chemiluminescence (ECL) kit (Merck Millipore, Billerica, MA, USA), as described in previous work [20].

### 4.6. ATPase Assays

The vanadate (Vi)-sensitive ATPase activities of ABCB1 and ABCG2 were assessed by measuring membrane vesicles obtained from High-Five insect cells (Invitrogen, Carlsbad, CA, USA) that were infected with recombinant baculovirus carrying the MDR1 gene or ABCG2 gene. The measurements were performed using the endpoint inorganic phosphate (Pi) assay, following the method described previously [33]. To determine the EC_50_ values, concentration-response curves were fitted based on the data obtained from at least three independent experiments. GraphPad Prism software version 5.0 (GraphPad Software, La Jolla, CA, USA) was used for this analysis, in accordance with the procedure described in previous work [19].

### 4.7. Docking Analysis

The chemical structure of furmonertinib, along with the inward-open cryo-EM structures of ABCB1 (PDB: 6QEX) [23] and ABCG2 protein (PDB: 8BI0) [24], underwent energy minimization using the CHARMM force field at pH 7.4. This procedure was carried out in BIOVIA Discovery Studio 4.0, following the previously described approach [48]. Subsequently, the docking of furmonertinib with both ABCB1 and ABCG2 was performed using the CDOCKER module within the same software.

### 4.8. Quantification and Statistical Analysis

The experimental values were presented as either mean ± standard error of the mean (SEM) or mean ± standard deviation (SD), calculated from at least three independent experiments, as indicated in the figure legends. Curve plotting was performed using GraphPad Prism software version 5.0 (GraphPad Software, La Jolla, CA, USA), and statistical analysis was carried out using KaleidaGraph software version 5.0 (Synergy Software, Reading, PA, USA). Statistical significance was determined by a two-tailed t-test, with a probability (*p*) value of less than 0.05 considered as indicative of a statistically significant difference.

## Figures and Tables

**Table 1:** Cytotoxicity of furmonertinib in human ABCB1- or ABCG2-overexpressing and parental cell lines.

Cell Line	Type	Transporter Overexpressed	IC_50_ [μM]^†^ (RF^‡^)
OVCAR-8	ovarian	-	1.77 ± 0.50 (1.0)
NCI-ADR-RES	ovarian	ABCB1	1.55 ± 0.25 (1.1)
KB-3-1	epidermal	-	2.57 ± 0.46 (1.0)
KB-V1	epidermal	ABCB1	2.77 ± 0.84 (1.1)
S1	colon	-	1.62 ± 0.34 (1.0)
S1-MI-80	colon	ABCG2	1.96 ± 0.52 (1.2)
H460	lung	-	2.58 ± 0.92 (1.0)
H460-MX20	lung	ABCG2	4.34 ± 1.73 (1.7)
pcDNA3.1-HEK293	-	-	3.20 ± 1.05 (1.0)
MDR19-HEK293	-	ABCB1	2.70 ± 0.75 (1.2)
R482-HEK293	-	ABCG2	3.33 ± 0.78 (1.0)

Abbreviation: RF, resistance factor. ^†^ IC_50_ values were calculated from dose-response curves obtained from at least three independent experiments, as described in Materials and Methods. ^‡^ RF values were obtained by dividing the IC_50_ value of furmonertinib in the ABCB1- or ABCG2-overexpressing multidrug-resistant cell lines by the IC_50_ value of furmonertinib in the corresponding drug-sensitive parental cell lines.

**Table 2:** Furmonertinib sensitizes ABCB1-overexpressing multidrug-resistant cells to cytotoxic drugs.

	Mean IC_50_^†^ ± SD and (FR^‡^)		
Treatment	Concentration (nM)	OVCAR-8 (Parental) [nM]	NCI-ADR-RES (Resistant) [nM]
Colchicine	-	23.90 ± 8.01 (1.0)	1526.20 ± 339.35 (1.0)
+ furmonertinib	50	22.75 ± 7.73 (1.1)	1250.47 ± 246.57 (1.2)
+ furmonertinib	100	23.57 ± 8.00 (1.0)	1074.86 ± 218.03 (1.4)
+ furmonertinib	200	21.34 ± 7.15 (1.1)	745.88 ± 149.48 * (2.0)
+ furmonertinib	500	20.22 ± 6.81 (1.2)	401.24 ± 88.79 ** (3.8)
+ tariquidar	1000	21.74 ± 7.40 (1.1)	19.93 ± 6.62 ** (76.6)
		[nM]	[nM]
Vincristine	-	13.58 ± 3.63 (1.0)	3686.23 ± 967.13 (1.0)
+ furmonertinib	50	13.65 ± 3.89 (1.0)	3167.10 ± 756.17 (1.2)
+ furmonertinib	100	11.61 ± 3.29 (1.2)	2301.36 ± 382.26 (1.6)
+ furmonertinib	200	10.94 ± 2.97 (1.2)	1406.00 ± 316.48 * (2.6)
+ furmonertinib	500	11.95 ± 3.72 (1.1)	353.57 ± 56.65 ** (10.4)
+ tariquidar	1000	12.75 ± 2.41 (1.1)	20.53 ± 4.91 ** (179.6)
		[nM]	[nM]
Paclitaxel	-	4.64 ± 1.39 (1.0)	6028.87 ± 1361.30 (1.0)
+ furmonertinib	50	5.56 ± 1.72 (0.8)	2112.46 ± 258.38 ** (2.9)
+ furmonertinib	100	4.81 ± 1.38 (1.0)	1517.14 ± 175.31 ** (4.0)
+ furmonertinib	200	4.74 ± 1.41 (1.0)	1151.65 ± 132.65 ** (5.2)
+ furmonertinib	500	4.38 ± 1.29 (1.1)	440.67 ± 69.20 ** (13.7)
+ tariquidar	1000	4.91 ± 1.71 (0.9)	6.81 ± 0.86 ** (885.3)
Treatment	Concentration (nM)	KB-3-1 (parental) [nM]	KB-V1 (resistant) [nM]
Colchicine	-	9.03 ± 3.56 (1.0)	1122.05 ± 88.87 (1.0)
+ furmonertinib	50	8.67 ± 3.53 (1.0)	1001.80 ± 67.14 (1.1)
+ furmonertinib	100	8.67 ± 3.56 (1.0)	883.44 ± 76.84 * (1.3)
+ furmonertinib	200	7.99 ± 3.04 (1.1)	709.68 ± 72.67 ** (1.6)
+ furmonertinib	500	7.93 ± 2.91 (1.1)	723.82 ± 117.88 ** (1.6)
+ tariquidar	1000	9.25 ± 3.58 (1.0)	13.90 ± 4.76 *** (80.7)
		[nM]	[nM]
Vincristine	-	0.61 ± 0.17 (1.0)	1326.18 ± 152.98 (1.0)
+ furmonertinib	50	0.70 ± 0.17 (0.9)	1454.11 ± 178.66 (0.9)
+ furmonertinib	100	0.85 ± 0.20 (0.7)	718.77 ± 86.65 ** (1.8)
+ furmonertinib	200	0.93 ± 0.19 (0.7)	341.47 ± 66.82 *** (3.9)
+ furmonertinib	500	0.70 ± 0.17 (0.9)	58.79 ± 7.72 *** (22.6)
+ tariquidar	1000	0.84 ± 0.21 (0.7)	2.46 ± 0.39 *** (539.1)
		[nM]	[nM]
Paclitaxel	-	2.20 ± 0.72 (1.0)	4853.95 ± 956.95 (1.0)
+ furmonertinib	50	2.50 ± 0.80 (0.9)	4135.80 ± 636.52 (1.2)
+ furmonertinib	100	2.28 ± 0.71 (1.0)	2559.00 ± 224.96 * (1.9)
+ furmonertinib	200	2.27 ± 0.67 (1.0)	856.05 ± 125.12 ** (5.7)
+ furmonertinib	500	1.94 ± 0.39 (1.1)	484.82 ± 35.84 ** (10.0)
+ tariquidar	1000	2.82 ± 0.95 (0.8)	2.01 ± 0.44 *** (2414.9)
Treatment	Concentration (nM)	pcDNA3.1-HEK293 (parental) [nM]	MDR19-HEK293 (resistant) [nM]
Colchicine	-	10.80 ± 3.65 (1.0)	252.60 ± 46.84 (1.0)
+ furmonertinib	50	13.46 ± 3.75 (0.8)	197.78 ± 28.41 (1.3)
+ furmonertinib	100	12.23 ± 3.30 (0.9)	199.57 ± 29.25 (1.3)
+ furmonertinib	200	11.70 ± 3.25 (0.9)	113.33 ± 15.25 ** (2.2)
+ furmonertinib	500	13.70 ± 3.28 (0.8)	85.80 ± 12.09 ** (2.9)
+ tariquidar	1000	10.48 ± 3.41 (1.0)	8.56 ± 2.86 *** (29.5)
		[nM]	[nM]
Vincristine	-	4.11 ± 0.88 (1.0)	622.69 ± 70.96 (1.0)
+ furmonertinib	50	3.55 ± 0.56 (1.2)	448.77 ± 48.45 * (1.4)
+ furmonertinib	100	2.91 ± 0.57 (1.4)	257.92 ± 40.13 ** (2.4)
+ furmonertinib	200	3.12 ± 0.67 (1.3)	145.20 ± 29.42 *** (4.3)
+ furmonertinib	500	3.41 ± 0.70 (1.2)	44.35 ± 10.68 *** (14.0)
+ tariquidar	1000	3.14 ± 0.72 (1.3)	3.95 ± 0.68 *** (157.6)
		[nM]	[nM]
Paclitaxel	-	2.14 ± 0.43 (1.0)	847.15 ± 90.57 (1.0)
+ furmonertinib	50	1.71 ± 0.23 (1.3)	1094.67 ± 104.23 * (0.8)
+ furmonertinib	100	2.23 ± 0.32 (1.0)	509.01 ± 67.50 ** (1.7)
+ furmonertinib	200	1.98 ± 0.26 (1.1)	335.46 ± 52.97 ** (2.5)
+ furmonertinib	500	2.04 ± 0.25 (1.0)	98.04 ± 15.98 *** (8.6)
+ tariquidar	1000	1.64 ± 0.37 (1.3)	2.57 ± 0.27 *** (329.6)

Abbreviation: FR, fold-reversal. ^†^ IC_50_ values were calculated from dose-response curves obtained from at least three independent experiments, as described in Materials and Methods. ^‡^ FR values were calculated by dividing the IC_50_ value of a known ABCB1 substrate drug by the IC_50_ value of the same substrate drug in the presence of furmonertinib or tariquidar. * *p* < 0.05; ** *p* < 0.01; *** *p* < 0.001.

**Table 3:** Furmonertinib sensitizes ABCG2-overexpressing multidrug-resistant cells to cytotoxic drugs.

		Mean IC_50_^†^ ± SD and (FR^‡^)	
Treatment	Concentration (nM)	S1 (Parental) [nM]	S1-MI-80 (Resistant) [nM]
Mitoxantrone	-	1.89 ± 0.26 (1.0)	85.12 ± 18.43 (1.0)
+ furmonertinib	50	2.48 ± 0.42 (0.8)	16.66 ± 2.83 ** (5.1)
+ furmonertinib	100	2.03 ± 0.25 (0.9)	8.12 ± 1.13 ** (10.5)
+ furmonertinib	200	1.51 ± 0.15 (1.3)	2.56 ± 0.46 ** (33.3)
+ furmonertinib	500	1.09 ± 0.11 ** (1.7)	0.42 ± 0.11 ** (202.7)
+ Ko143	1000	1.58 ± 0.21 (1.2)	0.64 ± 0.15 ** (133.0)
		[nM]	[nM]
SN-38	-	13.88 ± 1.14 (1.0)	2472.70 ± 432.26 (1.0)
+ furmonertinib	50	14.83 ± 3.33 (0.9)	1170.35 ± 190.54 ** (2.1)
+ furmonertinib	100	15.04 ± 3.52 (0.9)	264.30 ± 36.21 *** (9.4)
+ furmonertinib	200	12.15 ± 3.08 (1.1)	69.18 ± 8.10 *** (35.7)
+ furmonertinib	500	10.77 ± 3.45 (1.3)	32.95 ± 3.23 *** (75.04)
+ Ko143	1000	14.23 ± 1.05 (1.0)	25.26 ± 3.20 *** (97.9)
		[nM]	[μM]
Topotecan	-	80.77 ± 13.38 (1.0)	9.60 ± 0.89 (1.0)
+ furmonertinib	50	72.60 ± 11.15 (1.1)	2.42 ± 0.35 *** (4.0)
+ furmonertinib	100	73.24 ± 11.61 (1.1)	1.68 ± 0.20 *** (5.7)
+ furmonertinib	200	69.80 ± 11.95 (1.2)	0.30 ± 0.04 *** (32.0)
+ furmonertinib	500	69.18 ± 10.53 (1.2)	0.12 ± 0.02 *** (80.0)
+ Ko143	1000	70.82 ± 12.42 (1.1)	0.14 ± 0.02 *** (68.6)
Treatment	Concentration (nM)	H460 (parental) [nM]	H460-MX20 (resistant) [nM]
Mitoxantrone	-	2.87 ± 0.37 (1.0)	173.44 ± 31.97 (1.0)
+ furmonertinib	50	1.50 ± 0.19 ** (1.9)	53.51 ± 11.11 ** (3.2)
+ furmonertinib	100	0.89 ± 0.08 *** (3.2)	29.30 ± 5.41 ** (5.9)
+ furmonertinib	200	1.16 ± 0.15 ** (2.5)	18.75 ± 3.07 ** (9.3)
+ furmonertinib	500	0.80 ± 0.05 *** (3.6)	13.22 ± 2.15 *** (13.1)
+ Ko143	1000	1.67 ± 0.37 * (1.7)	14.68 ± 1.93 ** (11.8)
		[nM]	[nM]
SN-38	-	7.41 ± 0.70 (1.0)	122.85 ± 23.43 (1.0)
+ furmonertinib	50	6.21 ± 1.61 (1.2)	51.19 ± 11.39 ** (2.4)
+ furmonertinib	100	4.35 ± 0.80 ** (1.7)	28.49 ± 6.16 ** (4.3)
+ furmonertinib	200	4.09 ± 0.71 ** (1.8)	19.99 ± 4.00 ** (6.1)
+ furmonertinib	500	2.78 ± 0.38 *** (2.7)	11.25 ± 2.27 ** (10.9)
+ Ko143	1000	1.71 ± 0.21 *** (4.3)	4.00 ± 0.97 *** (30.7)
		[nM]	[nM]
Topotecan	-	37.38 ± 6.31 (1.0)	306.91 ± 93.67 (1.0)
+ furmonertinib	50	24.39 ± 3.44 * (1.5)	145.73 ± 38.23 (2.1)
+ furmonertinib	100	19.68 ± 3.37 * (1.9)	96.44 ± 27.03 * (3.2)
+ furmonertinib	200	20.86 ± 3.27 * (1.8)	56.66 ± 14.52 * (5.4)
+ furmonertinib	500	12.55 ± 1.67 ** (3.0)	45.34 ± 14.13 ** (6.8)
+ Ko143	1000	18.01 ± 2.58 ** (2.1)	14.23 ± 2.38 ** (21.6)
Treatment	Concentration (nM)	pcDNA3.1-HEK293 (parental) [nM]	R482-HEK293 (resistant) [nM]
Mitoxantrone	-	5.42 ± 0.60 (1.0)	336.14 ± 46.31 (1.0)
+ furmonertinib	50	4.38 ± 0.51 (1.2)	19.37 ± 2.08 *** (17.4)
+ furmonertinib	100	3.31 ± 0.41 ** (1.6)	12.12 ± 1.42 *** (27.7)
+ furmonertinib	200	3.00 ± 0.31 ** (1.8)	6.24 ± 0.92 *** (53.9)
+ furmonertinib	500	2.88 ± 0.38 ** (1.9)	5.82 ± 0.78 *** (57.8)
+ Ko143	1000	5.89 ± 0.54 (0.9)	7.74 ± 0.88 *** (43.4)
		[nM]	[nM]
SN-38	-	8.66 ± 2.00 (1.0)	635.73 ± 68.95 (1.0)
+ furmonertinib	50	13.04 ± 1.96 (0.7)	317.93 ± 26.51 ** (2.0)
+ furmonertinib	100	11.85 ± 1.69 (0.7)	160.41 ± 8.61 *** (4.0)
+ furmonertinib	200	11.81 ± 1.75 (0.7)	96.41 ± 10.31 *** (6.6)
+ furmonertinib	500	10.28 ± 1.49 (0.8)	48.84 ± 1.89 *** (13.0)
+ Ko143	1000	7.25 ± 1.67 (1.2)	13.50 ± 1.84 *** (47.1)
		[nM]	[nM]
Topotecan	-	99.39 ± 19.85 (1.0)	1579.30 ± 122.74 (1.0)
+ furmonertinib	50	99.82 ± 18.05 (1.0)	541.02 ± 36.31 *** (2.9)
+ furmonertinib	100	92.00 ± 14.52 (1.1)	400.12 ± 27.23 *** (3.9)
+ furmonertinib	200	97.31 ± 16.46 (1.0)	230.62 ± 19.11 *** (6.8)
+ furmonertinib	500	118.90 ± 18.70 (0.8)	116.67 ± 10.91 *** (13.5)
+ Ko143	1000	92.57 ± 18.20 (1.1)	154.15 ± 19.24 *** (10.2)

Abbreviation: FR, fold-reversal. ^†^ IC_50_ values were calculated from dose-response curves obtained from at least three independent experiments, as described in Materials and Methods. ^‡^ FR values were calculated by dividing the IC_50_ value of a known ABCB1 substrate drug by the IC_50_ value of the same substrate drug in the presence of furmonertinib or tariquidar. * *p* < 0.05; ** *p* < 0.01; *** *p* < 0.001.

## Data Availability

The data presented in this study are available on request from the corresponding author.
